# Identification and characterization of a novel metallo β-lactamase, SZM-1, in Shenzhen Bay, South China

**DOI:** 10.3389/fmicb.2022.996834

**Published:** 2022-09-26

**Authors:** Lingxu Fang, Zongbao Liu, Zhongyi Lu, Rongzhong Huang, Rong Xiang

**Affiliations:** ^1^Department of Stomatology, Shenzhen Qianhai Shekou Free Trade Zone Hospital, Shenzhen, China; ^2^Precision Medicine Center, The Second Affiliated Hospital of Chongqing Medical University, Chongqing, China; ^3^Key Laboratory of Ecology of Rare and Endangered Species and Environmental Protection, College of Life Sciences, Guangxi Normal University, Guilin, China; ^4^Archaeal Biology Center, Institute for Advanced Study, Shenzhen University, Shenzhen, China; ^5^Shenzhen Key Laboratory of Marine Microbiome Engineering, Institute for Advanced Study, Shenzhen University, Shenzhen, China

**Keywords:** metallo β-lactamase, metagenomic sequencing, carbapenem-resistance, SZM-1, carbapenemase

## Abstract

Metallo β-Lactamases (MBLs) degrade most clinical β-lactam antibiotics, especially Carbapenem, posing a huge threat to global health. Studies on environmental MBLs are important for risk assessment of the MBLs transmission among connected habitats, and between environment and human. Here, we described a novel metallo β-Lactamases, named SZM-1 (Shenzhen metallo-β-lactamase), from an *Arenimonas* metagenome-assembled genome recovered from the river sediment in the Shenzhen Bay area, south China. Phylogenetic analysis, primary sequence comparison, structural modeling suggested that the SZM-1 belongs to B1 MBL family, likely harboring a typical di-zinc catalytic center. Furthermore, the gene encoding the MBLs was cloned into *Escherichia coli* TOP10 for Carba NP test and antimicrobial susceptibility test. The results indicated that the SZM-1 had carbapenemase activity, and conferred the carrier to increased resistance toward carbapenems. Taken together, our results raise alarms about the emergence and spread of the SZM-1, and suggest further surveillance, especially in hospital settings and clinical isolates, to determine whether *bla*_SZM–1_ is a mobilizable antibiotic resistance.

## Introduction

Carbapenem is one of the last-resort β-lactam antibiotics for the treatment of serious bacterial infections that caused by gram-negative bacteria such as *Acinetobacter baumannii*, *Pseudomonas aeruginosa*, and *Klebsiella pneumonia*. Nevertheless, multiple molecular mechanisms that confer bacterial resistance phenotype become the major barrier to the clinical application of the drug (Kelly et al., 2017; Aurilio et al., 2022). As one of such mechanisms, metallo-β-lactamases (MBLs), can directly degrade β-lactam ring of the carbapenem and most other β-lactam antibiotics, through their zinc dependent catalytic active center (Abbas, 2021). Despite the development of efficient treatment options for infections caused by carbapenem-resistant Gram-negatives, such as ceftazidime-avibactam, meropenem-vaborbactam, imipenem-relebactam, and cefiderocol, only the later has demonstrated *in vitro* activity against MBL-producers (Mojica et al., 2022). Therefore, MBLs are still an alarming clinical problem, and the identification of new MBL genes important for understanding the epidemiology of this global threat.

To date, a majority of representative MBL types, for example NDM-1, IMP-1, VIM-1, and most their variants, have been firstly detected in clinical bacterial strains (Walsh et al., 1994; Lauretti et al., 1999; Poirel et al., 2000; Yong et al., 2009; Munita and Arias, 2016). While the wide distribution of the MBLs in cultured-based clinical isolates, increasing evidences have highlighted their environmental source, such as water environment (Deshmukh et al., 2011; Dziri et al., 2018; Fonseca et al., 2018). One reason for the close attention is that the environmental microorganisms represent a significant reservoir of novel antibiotic resistance genes (ARGs), including such MBL genes (Hernando-Amado et al., 2019). Besides, the transferable features of MBLs, which are mediated *via* mobile genetic elements, pose an urgent need for assessing the risk of the environment–human transmission (Bebrone, 2007).

Currently, metagenomic sequencing and analysis methods make it possible to globally monitor the MBL genes in a wide range of environments (Gudeta et al., 2016; Park et al., 2018; Hendriksen et al., 2019). For instance, several studies have used the metagenome-assembled genomes (MAGs) to identify bacterial hosts for the ARGs in both clinical and natural environments, providing new insights into the linkage of microbial community and the ARGs (Ma et al., 2016; Jia et al., 2019; Zhang et al., 2022). Moreover, methods for recovering MAGs from samples are considered important methodological extensions to traditional microbiological techniques and provide unprecedented access to uncultured organisms diversity, broadening the tree of life (Castelle and Banfield, 2018; Liu et al., 2021). Therefore, the metagenomic binning can contribute to a better understanding of the MBLs and provides important insights into the potential MBLs phylogenetic origin.

This study describes the identification of a novel subclass B1 MBL, SZM-1 (Shenzhen metallo-β-lactamase), in estuary sediment metagenomes from Shenzhen Bay area, one of the most developed areas with expanding population in south China (Yan et al., 2019).

## Materials and methods

### Metagenomic library construction

Three estuary sediment samples were collected at Dasha river of Shenzhen Bay in south China, and total DNA was extracted using the DNeasy PowerSoil Kit (Qiagen, Germany). Metagenomic sequence data were acquired using Illumina HiSeq sequencing with 150-bp paired-end reads at Novogene Bioinformatics Technology Co., Ltd. (Tianjin, China). The resulting raw sequencing reads were further dereplicated (100% identity over 100% length) and trimmed using sickle^1^. Remaining high-quality metagenomic reads were *de novo* metagenome assembled using MEGAHIT (Li et al., 2016) with parameters “–min-contig-len 1000 –k-min 21 –k-max 141 –k-step 12 –merge-level 20,0.95.” To perform gene prediction and annotation, the prokaryote annotation tool Prokka (Seemann, 2014) combined with the BLAST program^2^ was used.

### Identification of the SZM-1 in metagenome-assembled genomes

Metallo β-Lactamases were predicted in the metagenomic assemblies using ABRicate (v1.0.1)^3^ with the following thresholds: ≥70% DNA identity, ≥80% DNA coverage (Zankari et al., 2012). Our search for the putative MBLs obtained an IMP-1 like protein (share 81.03% sequence identify), designated SZM-1, encoding by an open reading frame of 699 bp (designated as *bla*_SZM–1_). Annotation of mobile elements was carried out using ISfinder^4^. To find out the bacterial host of the *bla*_SZM–1_, genome binning of the metagenome-assembled genomes (MAGs) was carried out using MetaBAT (Kang et al., 2019) with 12 sets of flags inducing different sensitivity and specificity combinations. CheckM (Parks et al., 2015) was used to calculate the completeness and contamination of MAGs. The ARG-carrying bin was selected for taxonomy classification with GTDB-Tk package (Chaumeil et al., 2019). The MAGs and *bla*_SZM–1_-carrying contigs were taxonomically classified using the default parameters of Kraken2 (Wood and Salzberg, 2014) and Kaiju (Tovo et al., 2020). Only *bla*_SZM–1_-carrying contigs that are longer than 10 kb were considered; taxonomic affiliation of the contig is agreed with the overall taxonomy of the MAG (Zhang et al., 2022).

### Bioinformatics analysis of the SZM-1

All the protein sequences were obtained from NCBI database,^5^ except the *bla*_SZM–1_ gene. Phylogenetic analysis was conducted using CLC Genomics Workbench. The protein sequences were aligned using MEGA7 (Kumar et al., 2016).

### *In silico* structure modeling of the SZM-1

The structure model for SZM-1 was built using the AlphaFold 2 of Colab server^6^. The multiple sequence alignment model, model type, pair mode, and the number of recycle were set as “UniRef + Environmental,” “auto,” “unpaired + paired,” and “3,” respectively. For each protein sequence, the one with the highest IDDT score of the resulting five models was used here.

### Cloning of the bla_SZM–1_ gene

The sequence of *bla*_SZM–1_ was codon optimized, synthesized (BGI Genomics Co., Ltd.) and cloned into a pHSG398 vector (without signal peptide) using restriction-free seamless cloning method. Briefly, the *bla*_SZM–1_ and the pHSG398 (TaKaRa Bio Co. Ltd., Japan) was amplified by PCR, respectively, using primers F-szm-1 (5′-ATGACCATGATTACGAATATGACCGCCGCAGGTGCAGA-3′)/R-szm-1 (5′-CCCGGGTACCGAGCTCGATTAATCATTC GGCAGCGGCGG-3′) and F-HSG (5′-TCGAGCTCGGTA CCCGGGGATC-3′)/R-HSG (5′-ATTCGTAATCATGGTCATA GCTG-3′) to construct the pHSG398-SZM-1. The resulting PCR fragments were purified and assembled with In-Fusion HD Cloning Kit (TaKaRa Bio Co. Ltd., Japan). To construct pHSG398-NDM-1, the full length of the NDM-1 (NCBI accession number: KX999121) was cloned into pHSG398 vector using restriction-free seamless cloning method with primers F-ndm-1 (5′- AGCTAT GACCATGATTACGAATATGCCGGGTTTCGGGGCAG-3′)/ R-ndm-1 (5′- CCGGGTACCGAGCTCGATCAGCGCAGCTT GTCGGCC-3′) and F-HSG (5′-TCGAGCTCGGTACCCGG GGATC-3′)/R-HSG (5′-ATTCGTAATCATGGTCATAGCTG-3′). Finally, the resulting recombinant plasmids were transformed into *Escherichia coli* TOP10 strains, respectively, that were used for further Carba NP test and resistance phenotype assay.

### Carba NP test

The Carba NP test was performed using the bacterial colonies according to the modified protocol as previously described (Pasteran et al., 2015).

### Antimicrobial susceptibility testing

Antimicrobial susceptibility testing was conducted using liquid broth dilution tests as recommended by CLSI guidelines (Clinical and Laboratory Standards Institute [CLSI], 2017). with Mueller-Hinton Broth (Oxoid Co. Ltd., UK). The *E. coli* TOP10 strains carrying the pHSG398 vector with a synthesized NDM-1 gene insert or no insert were used as positive and a negative control, respectively.

### Nucleotide sequence accession number

The metagenome-assembled genomes generated and analyzed during the current study are available in the NCBI (accession number: PRJNA848622).

## Results

### Identification of SZM-1 as a novel subclass B1 metallo-β-lactamase

**FIGURE 1:**
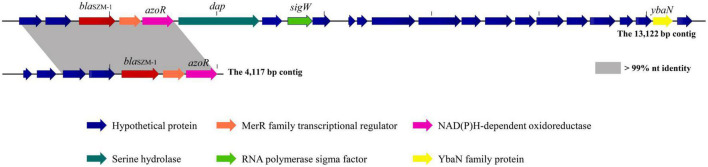
Genetic context surrounding the *bla*_SZM–1_ gene. The genetic contexts of *bla*_SZM–1_ on the 13,122-bp contig and the 4,117-bp contig are shown for comparison. The orfs that encode hypothetical proteins with unknown functions are shown in blue. Regions of >99% homology are marked with gray shading. The arrows indicate the positions and directions of transcription for each gene.

**FIGURE 2:**
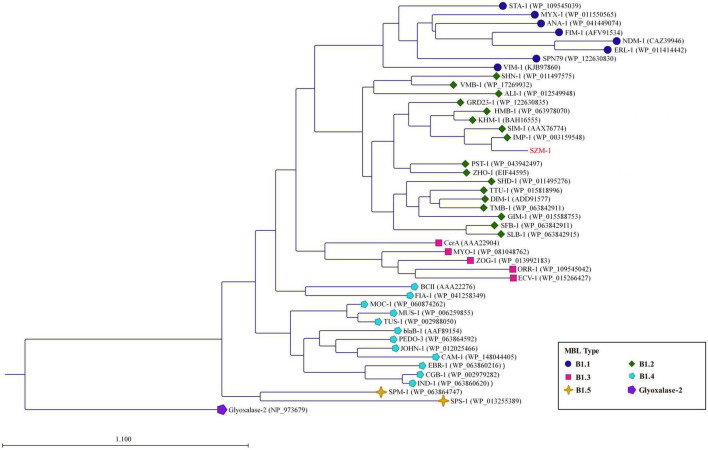
Phylogenetic analysis of the SZM-1. The phylogenetic tree was constructed with amino acid sequences of the representative MBL B1 subclasses (B1.1–B1.5) and Glyoxalase-2 (used as an outgroup). The tree was created by the Neighbor-Joining method (protein distance measure model: Jukes-Cantor; protein substitution model: WAG; bootstrap replicates: 1000). The horizontal bar indicates the number of amino acid substitution for site.

The genes encoding SZM-1 were identified in two assembled contigs with lengths of 13,122 and 4,117 bp, respectively (Figure 1). Except for *bla*_SZM–1_, other ORFs showed weak or no significant similarity to known sequences in NCBI-nr database. Binning analysis yielded a positive MAG for the *bla*_SZM–1_ gene, which consists of 89 contigs, including the 13,122 bp contig (genome completeness: 93.07%; contamination: 1.75%). In this MAG, no mobile genomic elements have been detected. Besides, the *bla*_SZM–1_-carrying MAG was taxonomically classified as genus *Arenimonas* within phylum Proteobacteria. The *bla*_SZM–1_ gene locates in a conserved genetic environment, no mobile element was identified in its vicinity (Figure 1), and the GC content of MAG (61.9%) and the *bla*_SZM–1_-carrying contig (65.7%) are at the same level. Meanwhile, the taxonomic affiliation of *bla*_SZM–1_-carrying contig agreed with the overall taxonomy of the MAG. Therefore, it can be concluded with high certainty that Arenimonas is the recent origin of *bla*_SZM–1_. Given that the SZM-1 shows high amino acid sequence identified to subclass B1 MBL1, and in order to phylogenetical analysis of the SZM-1, a maximum likelihood phylogenetic tree was constructed for the representative subclass B1 MBLs, SZM-1, and Glyoxalase-2 (used as an outgroup). As shown in the B1 MBLs tree, the SZM-1 was located close to the IMP-1 clade within the B1.2 branch; this tree topology clearly demonstrates that the SZM-1 belongs to subclass B1.2 MBLs (Figure 2). Nonetheless, analysis of amino acid sequence of the SZM-1 indicates the absence of the typical N-terminal signal peptide, implying the inefficient export of the novel MBL to periplasmatic space (Derman et al., 1993).

### Structural conservation of di-zinc catalytic center of SZM-1

**FIGURE 3:**
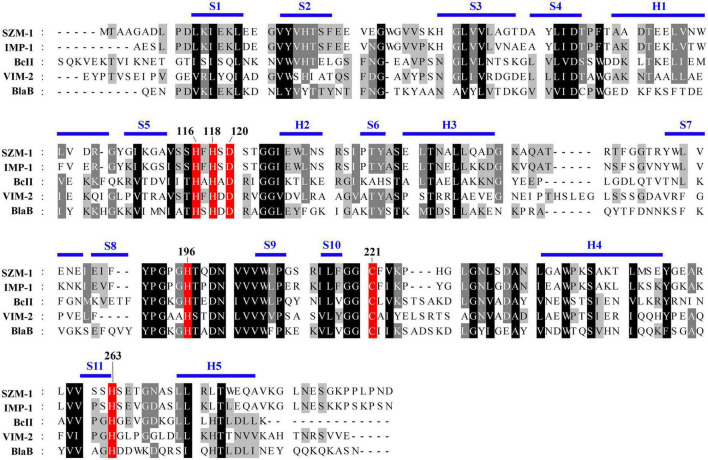
Amino acid sequence alignment of SZM-1 and the representative class B1 MBLs. The conserved class B residues are highlighted with red color. The representative class B1 MBLs include IMP-1 (NCBI accession number: WP 003159548.1), BcII (WP 000742468.1), VIM-2 (WP 003108247.1), and BlaB (WP 029729112.1). Conserved secondary-structure (S, β-strand; H, Helix) are indicated above the sequences. The key residues involved in the coordination of the zinc iron are numbered according to the standard MBL numbering system.

As previously described, the B1 MBLs have been characterized by mono-zinc or di-zinc dependent catalytic center for their enzymic activity (Bebrone, 2007; Llarrull et al., 2008). To characterize the zinc catalytic center of SZM-1, detailed sequence comparison of the SZM-1, as well as other B1 MBLs was performed. The primary sequence analysis revealed that the SZM-1 harbors conserved zinc-interacting residues that are well-characterized in B1 MBLs (Figure 3).

**FIGURE 4:**
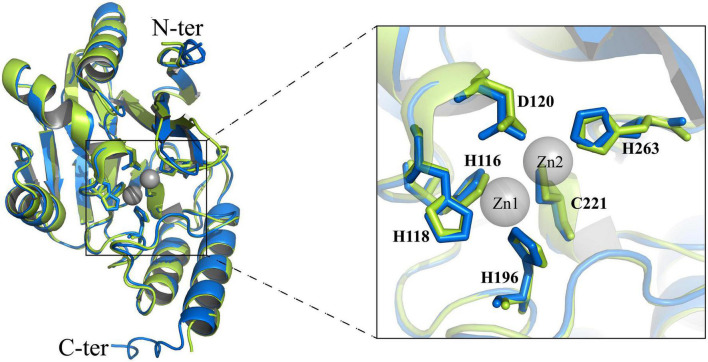
Structural analysis of the zinc-dependent active site of SZM-1. Structural comparison of active sites between IMP-1 (green) and SZM-1 (blue). The key residues (according to the standard MBL numbering system) involved in the coordination of the zinc iron are marked. The two zinc ions of the active site are marked by gray color.

Moreover, the structure of the full-length SZM-1 was predicted by the AlphaFold 2 of Colab server, and was further compared with the IMP-1 structure (PDB accession number: 1 ddk). The structural model display that the SZM-1 adopts a canonical αββα structure that resemble the IMP-1 structure with root-mean-square deviation (RMSD) value 0.446. Remarkably, according to the standard MBL numbering system, the conformation of the SZM-1 zinc-interacting active center, consisting of residues His116, His118, Asp120, His196, Cys221, and His263, is closely similar to that of the IMP-1 structure, strongly suggesting that its enzymatic activity is di-zinc dependent (Figure 4; Garau et al., 2004).

### Carbapenem-resistance phenotype of SZM-1

The *E. coli* TOP10 carrying *bla*_SZM–1_ showed carbapenemase activity by Carba NP test. Furthermore, when compared with the negative control, the *bla*_SZM–1_ carrier manifested a significant increase in MIC of Imipenem (>8-fold), while a slight increase of Meropenem (>2-fold; Table 1). In contrast, there was no difference in the MICs of aztreonam between the *bla*_SZM–1_ carrier and the negative control. Besides, expression of *bla*_SZM–1_ conferred *E. coli* TOP10 with reduced susceptibility to other tested β-lactams, including ampicillin (MIC = 64 mg/liter), cefotaxime (MIC = 4 mg/liter), ceftriaxone (MIC = 32 mg/liter), ceftazidime (MIC = 64 mg/liter), amoxycillin/clavulanic acid (MIC = 32 mg/liter), cefoxitin (MIC = 32 mg/liter), and piperacillin/tazobactam (MIC = 4 mg/liter). Together, these data reveal the carbapenem-resistance risks of the SZM-1.

**TABLE 1:** Antimicrobial drug susceptibility profile.

Antibiotic	MIC (mg/liter)		
	*E. coli* TOP10/pHSG398-NDM-1	*E. coli* TOP10/pHSG398-SZM-1	*E. coli* TOP10/pHSG398-
Ampicillin	>128	64	0.5
Cefotaxime	>128	4	0.25
Ceftriaxone	>128	32	0.25
Ceftazidime	>128	64	0.5
Amoxycillin/clavulanic acid	>128	32	0.25
Cefoxitin	>128	32	0.5
Piperacillin/tazobactam	>128	4	0.5
Imipenem	>64	2	<0.25
Meropenem	>64	0.5	<0.25
Aztreonam	<0.25	<0.25	<0.25

## Discussion

In this work, we reported a novel metallo β-lactamase, SZM-1, in Dasha river of Shenzhen Bay by shotgun metagenomic sequencing. The combined phylogenetic analysis, primary sequence alignment, and structural modeling indicate that the SZM-1 is a typical B1 MBL that likely employs a di-zinc dependent catalytic center. Moreover, these computational results are best compatible with the carbapenemase activity of the SZM-1, and the carbapenem-resistance phenotype of the *bla*_SZM–1_ carrier.

To the best of our knowledge, while the *Arenimonas* has been previously reported as a host of ARGs, this is the first-time characterization of a new MBL in this genus (Chen et al., 2019). Unlike the plasmid-borne IMP-1 and other mobile B1 MBLs, the absence of mobilizable elements in the genomic context of the *bla*_SZM–1_ seemingly does not contribute to the transmission of *bla*_SZM–1_ between organisms (Pongchaikul and Mongkolsuk, 2022). Nonetheless, given the foreign mobilizable elements may insert at genomic location around the *bla*_SZM–1_, transmission risk of such gene in hospital settings and other human living environments cannot be eliminated, especially when one notices the expanding population in Shenzhen Bay. Besides, the expansion of such MBLs has the potential to alter the structure of the bacterial population, leading to the emergence of antibiotic resistance environments (Hernando-Amado et al., 2019).

The MIC results indicated that reduce susceptibility of SZM-1 carrier to imipenem and meropenem, are comparable to that of IMP-1, which may be underpinned by the similar structures of di-zinc dependent catalytic center (Janda and Abbott, 2021). However, the SZM-1 displays reduced tolerance to β-lactam antibiotic, such as cefotaxime, suggesting the important roles of non-catalytic residues in the MBL activity. From a One Health perspective, the identification of the SZM-1 puts more emphasis on important roles of metagenomic analyses in the identification and detection of antibiotic resistance determinants from environmental microbiomes (Hernando-Amado et al., 2019). Our result also raised the awareness of the urgent for assessing the potential risk of novel MBLs in non-clinical ecosystems.

## Data availability statement

The datasets presented in this study can be found in online repositories. The names of the repository/repositories and accession number(s) can be found in the article/supplementary material.

## Author contributions

RX and RZH conceived, designed the study, and reviewed the manuscript. LF, ZbL, and ZyL performed the experiments, analyzed the data, and drafted the manuscript. All authors revised the manuscript and approved the final version.

## Footnotes

1
https://github.com/najoshi/sickle

2
http://blast.ncbi.nlm.nih.gov/Blast.cgi

3
https://github.com/tseemann/abricate

4
https://www-is.biotoul.fr/

5
https://www.ncbi.nlm.nih.gov

6
https://colab.research.google.com/github/sokrypton/ColabFold/blob/main/AlphaFold2.ipynb
